# Macronutrients in Human Milk and Early Childhood Growth—Is Protein the Main Driver?

**DOI:** 10.3390/nu16203514

**Published:** 2024-10-16

**Authors:** Jie Ma, Debra J. Palmer, Ching Tat Lai, Susan L. Prescott, Nina D’Vaz, Philip Vlaskovsky, Lisa F. Stinson, Zoya Gridneva, Donna T. Geddes

**Affiliations:** 1 School of Molecular Sciences, The University of Western Australia, Crawley, WA 6009, Australia; jie.ma@research.uwa.edu.au (J.M.); ching-tat.lai@uwa.edu.au (C.T.L.); lisa.stinson@uwa.edu.au (L.F.S.); donna.geddes@uwa.edu.au (D.T.G.); 2 ABREAST Network, Perth, WA 6000, Australia; debbie.palmer@uwa.edu.au; 3 UWA Centre for Human Lactation Research and Translation, Crawley, WA 6009, Australia; 4 The Kids Research Institute Australia, The University of Western Australia, Nedlands, WA 6009, Australia; susan.prescott@thekids.org.au (S.L.P.);; 5 School of Medicine, The University of Western Australia, Crawley, WA 6009, Australia; 6 Nova Institute for Health, Baltimore, MD 21231, USA; 7 Department of Family and Community Medicine, University of Maryland School of Medicine, Baltimore, MD 21201, USA; 8 School of Medical and Health Sciences, Edith Cowan University, Joondalup, WA 6027, Australia; 9 School of Mathematics and Statistics, The University of Western Australia, Crawley, WA 6009, Australia; philip.vlaskovsky@uwa.edu.au

**Keywords:** human milk macronutrients, infant intake, infant anthropometrics, infant growth, lactation, breastfeeding, protein leverage, lactose, fat, atopy

## Abstract

Background: Infant growth trajectories reflect current health status and may predict future obesity and metabolic diseases. Human milk is tailored to support optimal infant growth. However, nutrient intake rather than milk composition more accurately predicts growth outcomes. Although the role of protein leverage in infant growth is unclear, protein intake is important for early infancy growth. Materials and methods: This study of exclusively breastfeeding mothers with allergies (*n* = 161) from the Infant Fish Oil Supplementation Study assessed relationships between intake of human milk macronutrients and infant growth. Human milk fat, protein and lactose concentrations were measured at 3 months postpartum, and infant daily intakes were estimated using an average milk intake of 800 mL/day. Results: Higher human milk protein:energy ratio was associated with higher weight-for-age z-score at 2.5 years compared to 3 months and higher body mass index-for-age z-score change (6 months to 1 year compared to 3–6 months). Maternal atopy and birth season (summer) were negatively associated with human milk lactose concentration. Passive smoke exposure was associated with reduced energy and fat concentrations and increased lactose:energy ratio. Conclusions: Our results indicate that intake of human milk macronutrients may impact early childhood growth.

## 1. Introduction

Infant growth trajectory not only reflects current health status but may also predict future development of obesity and metabolic diseases [1,2,3]. Human milk is the optimal food source for infant growth and development, and contains a wide range of nutrients and bioactive components tailored specifically for human infants [4,5,6]. Recent studies suggest that analysis of measured infant intake is more accurate than studies that analyse only human milk component concentrations, as the former reflects actual nutrient consumption [7,8]. However, studies examining the relationship between infant intake of macronutrients and growth remain scarce.

As human milk-fed infants grow, the intake of milk per day per kilogram of body weight decreases, along with the concentration of protein in the milk and the total energy consumed [9,10]. Interestingly, the volume of human milk consumed by infants accounts for the greatest variability in growth [11]. Given constant infant milk intake, energy and macronutrient intakes in the first 6 months of life [12], the complexity of drivers of infant growth remain incompletely understood. Chronic overconsumption of macronutrients in adults leads to overweight and obesity [13]; however, an alternative hypothesis called the protein-leverage hypothesis suggests the overconsumption of fat and carbohydrates may be driven by inadequate protein intake [14,15,16]. The protein-leverage hypothesis posits that in a modern diet where protein is present at lower energy concentrations (such as high-fat and high-carbohydrate diet), individuals may overconsume total fat and carbohydrates to meet their individualised protein target. Protein leverage has been confirmed in both animal and adult human studies that have shown protein intake is more tightly regulated compared to fat and carbohydrates, particularly in diets low in protein and high in carbohydrates [14,17,18].

Although it is not clear when individual protein targets are programmed or whether protein leverage exists in breastfed infants, the fetus exhibits an exceeding rate of protein turnover that persists into the newborn period [19]. Low-weight preterm infants often require a higher protein intake of 3.5–4.0 g/kg/day, which can only be achieved from either fortification of human milk or formula feeding as these infants only tolerate small feed volumes [19,20]. Furthermore, the higher protein content in colostrum compared to mature milk [21,22] suggests that protein plays a crucial role in the early stages of an infant’s development.

This study aimed to explore associations between infant macronutrient intake during breastfeeding and early childhood growth in the first 2.5 years of life.

## 2. Materials and Methods

### 2.1. Study Population and Sample Collection

This is an exploratory analysis of data and human milk samples collected during the Infant Fish Oil Supplementation (IFOS) Study (Australian Clinical Trials Registry ACTRN12606000281594), which has been described elsewhere [23,24]. The IFOS Study was designed to assess the effects of infant fish oil supplementation on allergy and neurodevelopmental outcomes. All mothers of the participating infants had at least one allergic disease. In the intervention group, infants received 650 mg of fish oil daily, while the control group received 650 mg of olive oil daily. Fish oil supplementation did not impact allergy and neurodevelopmental outcomes [25,26]. Human milk samples were self-collected by 228 mothers at 3 months postpartum (93.9 ± 6.9 days) and transported to a −80 °C lab freezer until further analysis. A subset of 161 samples from fully breastfeeding mothers at 3 months was included in this analysis; of these, 42 infants were exposed to formula at some point prior to the establishment of full breastfeeding. Infant/child anthropometric parameters, including weight, length/height and head circumference, were collected in-person at the research clinic appointments at birth and at infant/child ages of 3 months, 6 months, 1 year and 2.5 years. Similarly, maternal characteristics and factors that may influence human milk macronutrients were recorded at the research clinic appointments in late pregnancy and at 3 months postpartum.

### 2.2. Macronutrient Measurements

Prior to further analysis, 1 mL of each milk sample was thawed in the 37 °C incubator for 30 min. Fat content was measured using the creamatocrit method [27]. Briefly, the percentage of the fat content was measured in a capillary tube after centrifugation at 12,000× *g* for 10 min at room temperature. Mean percentage of fat from 2 samples was used to calculate the concentration using the following equation [28]:

Total fat concentration (g/L) = (creamatocrit (%) − 0.59)/0.146,(1)

After fat measurement, the fat content was removed by cutting the capillary tube and the skimmed milk samples were obtained for protein and lactose analysis. The protein concentration in the skim milk was then measured using Bio-Rad DC Protein Assay Kit, adapted from the Lowry method [29], with human milk protein standard prepared according to the Kjeldahl method [30]. Assay recovery was 98 ± 2% and CV was 8.73%, with a detection limit of 0.079 g/L. The lactose concentration was measured using a colorimetric enzymatic assay (Megazyme lactose kit) [31]. This assay recovered 100.56 ± 6.58% in 8 sets, and CV was 6.55%. The detection limit of this assay was 5.824 g/L.

### 2.3. Calculation of Total Energy, Protein:energy Ratio and Estimated Macronutrient Intake

The estimated total energy was calculated from fat, protein and lactose using the following equation [32]:

Total energy concentration (kcal/L) = Total fat concentration (g/L) × 9 (kcal/g) + Total protein concentration (g/L) × 4 (kcal/g) + Total lactose concentration (g/L) × 4 (kcal/g)(2)

The estimated fat, protein and lactose intake was calculated on an average of 800 mL human milk consumption per day using the equation below [33,34]:

Total intake (g/day) = Total concentration (g/L) × 0.8 (L/day)(3)

Total energy intake was calculated using the equation below:

Total energy intake (kcal/day) = Total energy concentration (kcal/L) × 0.8 (L/day)(4)

Protein:energy ratio was calculated using the equation below:

% Protein:energy = Total protein concentration (g/L) ÷ Total energy concentration (kcal/L)(5)

### 2.4. Statistical Analysis

Descriptive statistics were reported as mean ± standard deviation (SD) and *n* (percentage), and modeling results as parameters estimate ± standard error (SE). Missing data were dealt with using available case analysis.

Linear mixed models (R function lmer) were fitted to the infant/child weight, length/height, body mass index (BMI) and head circumference z scores calculated from the Word Health Organisation [35] at 3 months, 6 months, 1 year and 2.5 years, as well as the changes in z scores from 3 to 6 months, from 6 months to 1 year and from 1 year to 2.5 years. For each infant outcome at each time point, the estimated intake of protein, fat, lactose and total estimated energy, infant age, as well as the energy ratios for protein, lactose and fat, and each of their interactions with infant age were included as explanatory variables, while participant ID was included as random effect. Anthropometric parameters at birth (weight/length/BMI/head circumference z scores), age when commenced solid food, age when stopped breastfeeding (breast milk vs. no breast milk), ever had infant formula by the age of 6 months (any volume), doctor diagnosed infant eczema by the age of 1 year, fish oil allocation group and compliance for fish/olive oil (percentage of capsules consumed) were included as confounders, as feeding practice and infant eczema have been shown to influence infant growth [36,37]. All continuous variables were centred and scaled prior to fitting into the models. Interactions with more than two levels (e.g., four time points of infant age) were examined using ANOVA to assess differences between these levels (e.g., whether the association between weight-for-age z score (WAZ) and protein intake at 1 year differs from that at 6 months). An association was considered significant only if both the *p*-value from the model and the *p*-value from the ANOVA were less than 0.05.

For the potential determinants of macronutrient concentration, linear models were fitted to each macronutrient variable, including concentrations of fat, protein and lactose, estimated total energy intake, fat:energy ratio, protein:energy ratio and lactose:energy ratio. Potential determinants include maternal ethnicity, maternal allergen sensitisation, maternal asthma, maternal hay fever, maternal food allergy, maternal eczema, delivery method, parity, passive smoke exposure during pregnancy, maternal antibiotics during breastfeeding at 3 months, infant fish oil intervention group, season of birth, infant sex, ever had formula by the age of 3 months and any furry pet at home at 3 months. ANOVA was performed for factors with more than two levels, including season of birth and maternal ethnicity. A *p*-value of <0.05 was considered significant for the factors with two levels and for continuous variables. For season of birth and maternal ethnicity, both the predictor *p*-value and the ANOVA *p*-value needed to be <0.05 to be considered significant.

For the association between macronutrient energy ratios, a power function was applied to analyse the relationships with total energy intake as below [38]:

Total energy intake (kcal) = P × *p^L^* (6)

where P represents a constant, *p* refers to the macronutrient energy ratios and *L* refers to the degree of leverage.

## 3. Results

**Figure 1:**
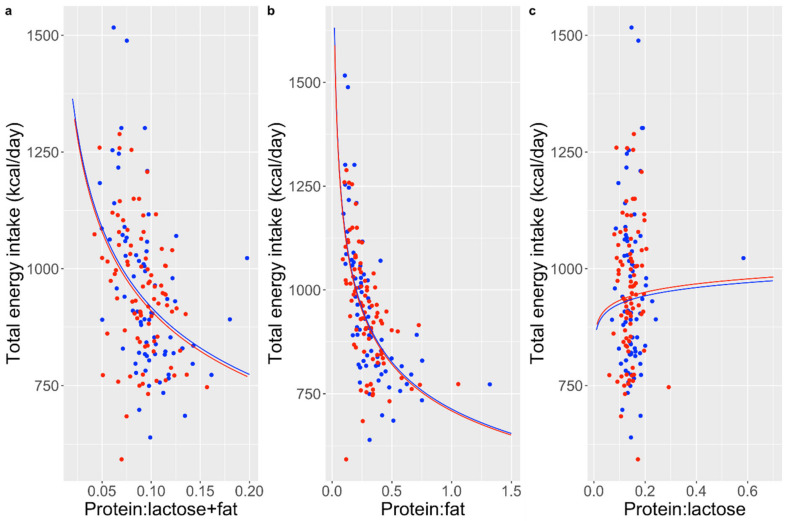
Relationships of macronutrient and energy ratios with total daily energy intake. (**a**) protein: fat + lactose; (**b**) protein:fat; (**c**) protein:lactose. Red colour indicates females and blue indicates males.

The maternal-infant dyad characteristics are recorded in Table 1 and Table 2. Macronutrient concentrations and estimated intakes are shown in Table 3. The average estimated intakes were similar to findings from previous studies that measured true infant intake over 24 h [8,39,40]. In this population of mothers with allergies, the proportion of energy from protein was relatively stable, while energy from fat and lactose was more variable. The protein:fat ratio, and protein:sum of fat plus lactose ratio, were negatively associated with total energy intake, whereas there was no association with the protein:lactose ratio (Figure 1).

Among the 18 potential determinants analysed, 3 were significantly associated with the macronutrient content of human milk (Table 4). Milk of mothers who were sensitised to common allergens had reduced concentrations of lactose (*p* = 0.0497). Notably, reduced lactose concentration was also associated with summer birth season (*p* = 0.033). Milk of mothers who were not exposed to passive smoke during pregnancy had higher total energy and fat concentrations (*p* = 0.022, *p* = 0.025, respectively), as well as reduced lactose:energy ratio (*p* = 0.046).

Infant z scores at birth were significantly associated with the corresponding growth parameters up to 2.5 years of age, but not with the changes between time points, except for the changes in WAZ (Table A1). Additionally, the age when breastfeeding stopped was negatively associated with change in WAZ (−0.019 (0.006), *p* = 0.002) and change in length-for-age z score (LAZ, −0.0008 (0.0003), *p* = 0.018). Also, infants with a history of formula exposure by the age of 3 months showed a greater change in WAZ (0.20 (0.088), *p* = 0.023). Infants diagnosed with eczema by the age of 1 year had significantly lower WAZ (−0.034 (0.015), *p* = 0.028). Infants with better adherence to daily fish/olive oil supplementation had significantly lower BMI-for-age z score (BMIAZ, −0.0005 (0.0002), *p* = 0.032).

The only macronutrient in human milk associated with early childhood growth by the age of 2.5 years was the protein:energy ratio (Table 5). Specifically, a higher protein:energy ratio was associated with a greater change in BMIAZ from 6 months to 1 year compared to 3 to 6 months change (*p* = 0.023). Additionally, a higher protein:energy ratio was associated with a higher WAZ at 2.5 years compared to 3 months (*p* = 0.015). 

## 4. Discussion

In this study, the positive associations between protein:energy ratio and infant/child growth parameters highlight the possibility of partial protein leverage in breastfed infant growth. The protein-leverage concept suggests that humans at least partially prioritise protein consumption over other macronutrients. A randomised controlled trial found that lowering dietary protein from 15% to 10% led to a significant increase in total energy intake, but increasing protein to 20% had no effect, indicating low protein levels may trigger overeating of other nutrients [41]. Similar results have been observed in other studies, including among adolescents, where high-protein diets resulted in lower energy intake compared to low-protein diets [42,43].

Whilst there is relatively strong evidence for protein leverage in adults and adolescents, it is not known if protein targets are inherited or developed in early infancy. Evidence exists to suggest that increased human milk protein intake is associated with increased weight gain and BMI [44], although actual intake was not measured and the associations were only significant for the growth from birth to 6 weeks. Similarly, increased protein intake in our study was associated with increased LAZ and WAZ at 2.5 years compared to 3 months (prior to ANOVA). More importantly, we found that increased protein:energy ratios were related to increased WAZ at 2.5 years compared to those at 3 months, as well as change in BMIAZ from 6 months to 1 year compared to the change from 3 to 6 months. This is somewhat aligned with contemporary guidelines recommending increased protein:energy ratios of preterm infant feeds to their high energy requirement for growth and development [19]. Our findings require further investigation in larger studies where infant human milk macronutrient intake will be measured.

In our study, human milk lactose was estimated to provide 43.5% of energy to the infant yet lactose intake or the lactose:energy ratio was not associated with any growth parameters. Although filtered out by a borderline ANOVA *p*-value, we found a negative correlation between lactose intake and change in BMIAZ from 1 year to 2.5 years. In a cross-sectional study, lactose intake at 3 months of age was positively associated with infant weight and length at 3 months [40]. A small study (*n* = 94) found that, whilst higher lactose intake at 6 weeks of age was associated with increased gain in weight and BMI from 0 to 3 months, the associations were negative for weight and length gains from 3 to 12 months [44]. Another study (*n* = 20) showed a similar pattern where the relationships of lactose intake with infant BMI and fat-free mass was positive at up to 9 months and negative at 12 months of age, whereas a relationship with fat mass remained positive [8]. The initial increase and then decrease (around 5–9 months) and stabilisation of the infant fat tissue accrual in comparison to continuing increase of the fat-free mass [45] could explain these temporal relationships. In addition to energy source, lactose also serves as prebiotic for the infant gut microbiota and potentially could impact infant development via the modification of the microbial structure in the gut [46,47]. Given lactose concentration has been shown to decrease as lactation progresses [48], further research may clarify these time-dependent associations.

We found no relationships between human milk fat intake or fat:energy ratio and growth over the first 2.5 years of life. Studies investigating relationships between fat concentration and infant growth document conflicting results, with most reporting no associations and others reporting positive or negative associations [7,49]. A single longitudinal study has measured fat intake and reported that infants (*n* = 13) with high weight gain received more fat from human milk at 3 months of age than those with normal weight gain (*n* = 15) [39]. Whilst in our study fat contributed 50.3% to energy content this was also balanced by lactose contributing a similar percentage (43.5%). Further, fat concentrations do not accurately reflect intake as they vary with the degree of fullness of the breast, after milk removal and across the day. Well-designed studies with multiple sampling strategies are required to improve our understanding of the contribution of human milk total fat to infant growth and body composition.

Another novel finding of this study is the relationship of maternal atopy with increased human milk lactose concentration. This is interesting as an in vitro study has shown that synthesis of lactose in lactating mammary epithelial cells is inhibited by pro-inflammatory cytokines TNF-α, IL-1β and IL-6, all of which play roles in allergy [50]. Furthermore, lactose concentration was lower in mothers who gave birth in summer compared to those who gave birth in spring. This difference may be related to the peak of seasonal allergies, such as to grass pollen, in spring in Perth (Australia), as all mothers in this study had allergic disease(s). However, these results should be interpretated with caution as 94.4% of these mothers had atopy, leading to unbalanced statistical power and reduced reliability. Nevertheless, these results are of interest given the limited knowledge on how maternal allergic diseases may impact infants through breastfeeding from the developmental origins of health and disease perspectives.

Additionally, our observed negative associations between passive smoke exposure and reduced human milk energy and fat concentrations align with existing literature showing smoke exposure is related to lower human milk total lipid levels [51,52,53,54]. Given it was not associated with lactose concentration, the negative association with lactose:energy ratio is likely driven by fat. This association with fat in human milk is likely because nicotine influences lipoprotein lipase of human adipose tissue, an enzyme involved in breaking down triglycerides in adipose tissue [51]. It may also affect enzymes and proteins that regulate lipid transport and metabolism, suggesting alteration in maternal lipid metabolism, particularly the mammary gland, which subsequently influences lactogenesis [51,54].

While the majority of current human milk intake studies have recorded short-term growth data up to age of 12 months, our study was able to track infant/child growth up to 2.5 years [8,39,40,44]. Limitations included estimation of macronutrient intakes, although based on published reference milk intake data, as well as the absence of body composition data, which restricted our analysis to anthropometric measurements alone.

## 5. Conclusions

In this study of infants born to mothers with allergic disease(s), human milk protein was associated with increased growth, whilst human milk lactose exhibited a negative association. Further studies are required to elucidate contributors to early infant growth in the context of potential programming for later risk of overweight and obesity.

## Figures and Tables

**Table 1:** Maternal and infant characteristics.

Characteristics	
Maternal age (years) (*n* = 161)	33.2 ± 4.1 ^a^
Parity (*n* = 161)	1.8 ± 0.9
Ethnicity (*n* = 157)	
Caucasian	146 (93.0%)
Asian	7 (4.5%)
Other	4 (2.5%)
Birth gestation (weeks) (*n* = 150)	39.2 ± 1.2
Maternal atopy (*n* = 161)	152 (94.4%)
Age breastfeeding stopped (months) (*n* = 151)	11.9 ± 5.4
Age solid foods commenced (months) (*n* = 154)	5.4 ± 0.7
Infant eczema diagnosed by age of 1 year (*n* = 147)	93 (63.3%)

^a^ Data are presented as mean ± SD or *n* (%).

**Table 2:** Infant anthropometrics.

Parameters	Birth	3 Months	6 Months	1 Year	2.5 Years
Weight (kg)	3.47 ± 0.42 ^a^	6.25 ± 0.75	7.78 ± 0.88	9.91 ± 1.12	14.1 ± 1.68
WAZ	0.058 ± 0.13	0.027 ± 0.11	0.026 ± 0.11	0.071 ± 0.11	0.088 ± 0.13
Length/height (cm)	50.6 ± 2.28	61.0 ± 2.25	66.8 ± 2.19	76.0 ± 3.26	92.7 ± 4.02
LAZ	0.022 ± 0.046	0.007 ± 0.036	0.003 ± 0.031	0.016 ± 0.043	0.015 ± 0.044
HC (cm)	34.9 ± 1.94	41.1 ± 1.47	43.8 ± 1.41	46.8 ± 1.48	50.1 ± 1.58
HCAZ	0.021 ± 0.057	0.029 ± 0.033	0.025 ± 0.029	0.031 ± 0.028	0.036 ± 0.030
BMI (kg/m^2^)	13.6 ± 1.43	16.8 ± 1.54	17.4 ± 1.47	17.2 ± 1.35	16.4 ± 1.38
BMIAZ	0.016 ± 0.110	0.014 ± 0.089	0.018 ± 0.084	0.038 ± 0.078	0.050 ± 0.087

^a^ Data are presented as mean ± SD. BMI, body mass index; BMIAZ, BMI for age z score; HC, head circumference; HCAZ, head circumference for age z score; LAZ, length/height for age z score; WAZ, weight for age z score. Weight and WAZ at birth *n* = 161, at 3 months *n* = 157, at 6 months *n* = 146, at 1 year *n* = 138, at 2.5 years *n* = 125; Length/height and LAZ at birth *n* = 161, at 3 months *n* = 157, at 6 months *n* = 146, at 1 year *n* = 140, at 2.5 years *n* = 127; HC and HCAZ at birth *n* = 156, at 3 months *n* = 154, at 6 months *n* = 141, at 1 year *n* = 137, at 2.5 years *n* = 122; BMI and BMIAZ at birth *n* = 161, at 3 months *n* = 157, at 6 months *n* = 146, at 1 year *n* = 138, at 2.5 years *n* = 125.

**Table 3:** Human milk macronutrient concentrations and intakes.

Macronutrients	Concentration (g or kcal/L)	Intake (g or kcal/Day)
Protein	11.0 ± 2.55 ^a^	8.77 ± 2.04
Fat	43.6 ± 20.3	34.9 ± 16.2
Lactose	76.9 ± 9.50	61.5 ± 7.60
Energy	744 ± 184	595 ± 147
Protein:energy ratio	-	6.16% ± 1.81%
Fat:energy ratio	-	50.3% ± 11.8%
Lactose:energy ratio	-	43.5% ± 10.6%

^a^ Data are presented as mean ± SD.

**Table 4:** Factors that influence the macronutrients in human milk.

Macronutrients	Predictors	Estimate	SE	Predictor *p*-Value ^b^	ANOVA *p*-Value ^c^
Energy	Not exposed to passive smoke	75.276 ^a^	32.522	0.022	-
Fat	Not exposed to passive smoke	8.035	3.536	0.025	-
Fat:energy ratio	Birth season summer	0.060	0.030	0.046	0.177
Lactose	Birth season summer	−5.400	2.506	0.033	0.029
	Maternal atopy	−8.851	4.468	0.050 *	-
Lactose:energy ratio	Not exposed to passive smoke	−0.037	0.018	0.046	-
Protein:energy ratio	Birth season autumn	−0.010	0.005	0.031	0.084
	Birth season summer	−0.010	0.005	0.038	0.084

^a^ Data are parameter estimate and standard error (SE); ^b^ the predictor *p*-value indicates the significance of individual predictors in the linear mixed model output; ^c^ the ANOVA *p*-value indicates the significance from the analysis of variance; * *p* = 0.04974 rounded up to 0.05. For predictors with more than 2 levels (birth season), only if both predictor and ANOVA *p*-values are less than 0.05 should the results be considered significant.

**Table 5:** Associations between the human milk macronutrients and infant growth.

Infant Growth Parameters	Time	Macronutrients	Estimate	SE	Predictor *p*-Value ^d^	ANOVA *p*-Value ^e^
ΔBMIAZ	6 months to 1 year ^b^	Protein:energy ratio	0.021 ^a^	0.009	0.023	0.047
	1 year to 2.5 years ^b^	Lactose intake	−0.027	0.012	0.025	0.051
	1 year to 2.5 years ^b^	Energy intake	−0.021	0.010	0.031	0.054
	1 year to 2.5 years ^b^	Fat intake	−0.020	0.010	0.043	0.096
	6 months to 1 year ^b^	Protein intake	0.019	0.010	0.046	0.094
HCAZ	1 year ^c^	Fat energy ratio	0.005	0.002	0.027	0.178
	1 year ^c^	Lactose energy ratio	−0.005	0.002	0.028	0.182
	1 year ^c^	Energy intake	0.005	0.002	0.030	0.186
LAZ	2.5 years ^c^	Protein intake	0.007	0.003	0.032	0.192
	2.5 years ^c^	Protein:energy ratio	0.006	0.003	0.045	0.212
WAZ	2.5 years ^c^	Protein:energy ratio	0.021	0.009	0.015	0.022

^a^ Data are parameter estimate and standard error (SE); ^b^ baseline is a change from 3 months to 6 months; ^c^ baseline is 3 months; Δ indicates change between the time point and the baseline. ANOVA, analysis of variance; BMIAZ, BMI for age z score; HCAZ, head circumference for age z score; LAZ, length/height for age z score; WAZ, weight for age z score. ^d^ The predictor *p*-value indicates the significance of individual predictors in the linear mixed model output; ^e^ the ANOVA *p*-value indicates the significance from the analysis of variance; only if both predictor *p*-value and ANOVA *p*-value are less than 0.05 should the results be considered significant.

## Data Availability

Restrictions apply to the availability of some, or all data generated or analysed during this study. The corresponding author will on request detail the restrictions and any conditions under which access to some data may be provided.

## Appendix A

**Table A1:** Associations between birth z scores and the z scores and changes in z scores after birth.

Anthropometrics from 3 Months to 2.5 Years ^b^	Birth z Scores	Estimate	SE	*p*-Value
BMIAZ	BMIAZ	0.1590 ^a^	0.055	0.004
ΔBMIAZ	BMIAZ	−0.016	0.040	0.682
HCAZ	HCAZ	0.205	0.035	<0.001
ΔHCAZ	HCAZ	−0.010	0.023	0.668
LAZ	LAZ	0.192	0.057	0.001
ΔLAZ	LAZ	−0.048	0.038	0.215
WAZ	WAZ	0.354	0.061	<0.001
ΔWAZ	WAZ	0.931	0.271	0.001

^a^ Data are parameter estimate and standard error (SD). BMI, body mass index; BMIAZ, BMI for age z score; HC, head circumference; HCAZ, head circumference for age z score; LAZ, length/height for age z score; WAZ, weight for age z score. ^b^ For BMIAZ, HCAZ, LAZ and WAZ, time points include 3 months, 6 months, 1 year and 2.5 years; for ΔBMIAZ, ΔHCAZ, ΔLAZ and ΔWAZ, time points include changes from 3 to 6 months, from 6 months to 1 year and from 1 to 2.5 years. In the linear mixed models, all time points were included with participant as a random effect.
